# Haemosporidian and trypanosomatid diversity in a high-latitude island ecosystem, including the first record of *Zelonia* in the Nearctic

**DOI:** 10.1007/s00436-025-08490-4

**Published:** 2025-05-01

**Authors:** Jacqueline Savage, Jaclyn Santos, Paul R. Sweet, Spencer C. Galen

**Affiliations:** 1 Biology Department, University of Scranton, Loyola Science Center, Scranton, PA 18510 USA; 2 Department of Ornithology, American Museum of Natural History, New York, NY 10024 USA

**Keywords:** Alaska, Biodiversity, Haemosporidian, Island, *Trypanosoma*, *Zelonia*

## Abstract

Biodiversity surveys remain a critical tool for characterizing the global species richness of parasites. In high-latitude regions of the world, characterizing parasite biodiversity is of particular importance due to the rapid rate at which the climate is changing and potentially shifting parasite distributions and abundances. We sampled a bird community on Prince of Wales Island in southern Alaska, United States, to test for the abundance and richness of haemosporidian and trypanosomatid parasites in this understudied region. We tested for parasites in 67 archived tissue samples of 18 bird species, of which five had not been previously tested for haemosporidians and 11 had not been sampled for trypanosomatids using molecular methods. We recovered two novel mitochondrial genetic lineages of haemosporidians (genera *Haemoproteus* and *Leucocytozoon*), and three novel 18S rRNA genotypes belonging to *Trypanosoma* of uncertain species-level affinities. Surprisingly, we also identified a trypanosomatid from the genus *Zelonia*, a group of monoxenous parasites of insects, from an avian tissue. While this anomalous record may have been the result of environmental contamination, it nonetheless reflects the first record of *Zelonia* in the entire Nearctic region.

**Supplementary Information:**

The online version contains supplementary material available at 10.1007/s00436-025-08490-4.

## Introduction

The global biodiversity of parasites is likely to still be largely undiscovered (Poulin 2014, Okamura et al. 2018, Carlson et al. 2020). At a basic level, a major obstacle in the way of understanding the global diversity of parasites is the simple fact that most regions of the world and most potential host groups have not been surveyed for the myriad parasite groups in existence (Poulin 2014). Although the formal description of parasite species and deposition of specimens in biodiversity repositories is the gold standard for documenting global parasite biodiversity, rapid methods of biodiversity discovery have an important role in the simple recognition of the existence of undescribed parasite diversity (Besansky et al. 2003). Approaches such as the use of molecular methods to detect distinct genetic lineages of morphologically cryptic or difficult to study parasite groups is especially important in this regard.

Globally, high-latitude regions have recently become the focus of increasing interest in parasitology due in part to the anticipated dramatic effects of climate change (Kutz et al. 2005, Dobson et al. 2015). For instance, due to the increasing global temperatures in northern latitudes and higher altitudes, the transmission area of some parasites is likely to change (Kutz et al. 2005, Loiseau et al. 2012). Furthermore, the introduction of new parasites into high-latitude areas through altered host movements due to climate change may result in severe ecological impacts in naïve hosts (Atkinson & LaPointe 2009, Kutz et al. 2013). Given documented shifts in animal distributions across high-latitude regions (Brommer et al. 2012, Mizel et al. 2016), researching the parasites and hosts that currently inhabit these areas is critical for characterizing future change.

In recent years, the high-latitude forests of North America have been demonstrated to be a hotbed of undiscovered blood parasite genetic diversity infecting birds (Oakgrove et al. 2014, Reeves et al. 2015, Smith et al. 2016, Galen et al. 2019, De Amaral et al. 2023). Parasites in the order Haemosporida, including *Haemoproteus*, *Leucocytozoon*, and *Plasmodium*, have been shown to be highly abundant and diverse across high-latitude birds in North America, including Alaska. For instance, in a single bird species (*Corvus caurinus*) sampled at six sites in southeastern Alaska, Van Hemert et al. (2019) discovered 10 distinct haemosporidian lineages, with *Leucocytozoon* being most prevalent and diverse. However, the high-latitude forests of North America are still broadly under sampled for haemosporidians relative to the host and habitat diversity that exists in this region. In fact, there has been no general survey of haemosporidians in the region of southeastern Alaska, where the largest National Forest in the United States, Tongass National Forest, is located.

In addition to haemosporidians, organisms in the family trypanosomatidae (trypanosomes and their relatives) are abundant and diverse parasites of wildlife globally with great potential for biodiversity discovery (Maslov et al. 2013, Chandler and James 2013, Cottontail et al. 2014). The genus *Trypanosoma* is among the most intensively studied of the trypanosomatids due to the pathogenicity of some members of this genus, yet like the haemosporidians, the application of molecular approaches has revealed previously unrecognized species-level diversity in this genus (Lima et al. 2013, Lemos et al. 2015, Mafie et al. 2019, Votýpka et al. 2022). Yet unlike the haemosporidians, very few sites and host species globally have been surveyed for trypanosomes using molecular techniques. Interestingly, birds have been infrequently studied for trypanosomes while simultaneously avian haemosporidian research has become increasingly common over the last 25 years.

While genera such as *Trypanosoma* and *Leishmania* are well-studied due to their potential to cause disease in vertebrates, there are many poorly known trypanosomatid genera that have recently begun to receive increased attention (Lukeš et al. 2018). One such trypanosomatid is the genus *Zelonia*, a group of monoxenous parasites of insects that is a sister lineage to the clade that contains the genus *Leishmania* (Espinosa et al. 2018, Barratt et al. 2017). *Zelonia* has been isolated from insects in the orders Hemiptera and Diptera largely in warm, tropical regions of the southern hemisphere (Yurchenko et al. 2006, Barratt et al. 2017, Votýpka et al. 2020). However, Malysheva et al. (2023) discovered *Zelonia daumondi* in Russia, representing the first record of *Zelonia* for the Holarctic and the highest latitude record to date. This surprising finding suggests that our understanding of the diversity and distribution of *Zelonia* and other poorly studied trypanosomatids is still in its infancy, and further surveys in temperate and boreal regions of the world are likely to uncover previously unknown species diversity.

In this study, we report on a molecular survey of haemosporidians and trypanosomatids isolated from birds from a high-latitude island site in North America that has not previously been studied for these parasites. We report novel haemosporidian and trypanosomatid genetic diversity from previously unsurveyed hosts, including a record of *Zelonia* that substantially expands our knowledge of the geographic range of this understudied genus.

## Methods

### Sample collection

We used avian tissue (muscle and liver) samples archived in the American Museum of Natural History to test for the presence of haemosporidians and trypanosomatids. These samples were collected from 67 individuals of 18 species of bird sampled from the Tongass National Forest on Prince of Wales Island in Alaska, United States in June 2019 (Supplementary Appendix). Prince of Wales Island is the largest island of the Alexander Archipelago (6670 km^2^), consisting predominantly of temperate rainforest. Prince of Wales Island has a distinct terrestrial fauna, characterized by several vertebrate subspecies that are endemic to the island (Barry and Tallmon 2010).

Tissue samples were dissected from avian hosts during the process of specimen preparation in the field, and were immediately placed into RNALater for long-term preservation at − 20 °C. Samples were collected according to permit 19–150 from the State of Alaska Department of Fish and Game, and permit MB779035 - 0 from the U.S. Fish and Wildlife Service.

### Molecular detection of haemosporidians and trypanosomatids

DNA was extracted from approximately 25 mg of the liver and muscle tissue samples using the New England BioLabs Monarch Genomic DNA Purification Kit, following the solid tissue sample protocol with 100-μL final elution buffer. Polymerase chain reaction (PCR) was performed to amplify gene fragments for both haemosporidians and trypanosomatids. First, we used a nested PCR protocol (Hellgren et al. 2004) to test for the presence of haemosporidian parasites of the genera *Haemoproteus*, *Plasmodium*, and *Leucocytozoon*. This protocol amplifies a 479 base pair fragment of the cytochrome b gene (*cytb*) and has been used as a standard for the molecular detection of avian haemosporidians for the last 20 years (Hellgren et al. 2004). The outer reaction was performed using primers HaemNFI and HaemNR3, and 1 µL of the outer reaction product was used as the template for two inner reactions, one using primers HaemF and HaemR2 (amplification of *Haemoproteus* and *Plasmodium*), and the other using primers HaemFL and HaemR2L (amplification of *Leucocytozoon*). Details of the reactions and cycling conditions followed Hellgren et al. (2004).

Trypanosomatids were detected using a nested PCR protocol that targets a 770 base pair fragment of the 18S rRNA gene following Valkiūnas et al. (2011). Briefly, this protocol uses outer primers Tryp763 and Tryp1016 for an initial PCR, using 1 µL of this reaction as the template for an inner reaction using primers Tryp99 and Tryp957. Cycling conditions for outer and inner reactions followed Valkiūnas et al. (2011). For all PCRs, agarose gel electrophoresis was used to identify amplifications using SYBR Safe (Invitrogen) stain. All samples that were positive for haemosporidian or trypanosomatid infection were sent to GENEWIZ by Azenta Life Sciences (South Plainfield, New Jersey) for Sanger sequencing.

### Sequence analysis

Each sequence was assembled and edited using the program Geneious Prime v2023.2. For haemosporidians, the genus and genetic lineage of each sequence were determined using a custom Basic Local Alignment Search Tool (BLAST) database using the sequences available from the MalAvi online database (Bensch et al. 2009). New genetic lineages were defined as sequences with at least one DNA substitution relative to a preexisting sequence in the MalAvi database. The new lineages were named in accordance with MalAvi haemosporidian naming conventions, using a six-letter code consisting of the first three letters of the host genus and the first three letters of the host species, followed by a unique number. Haemosporidian coinfections were identified by the presence of multiple distinct peaks in sequence chromatograms. We followed the approach described by Starkloff and Galen (2023) to manually separate coinfecting haemosporidian lineages.

As there is no centralized repository for avian trypanosomatid 18S sequences, we used BLAST searches to determine whether our newly generated sequences have been detected previously and have been deposited in GenBank.

### Phylogenetic analyses

We estimated a phylogenetic tree for all haemosporidian lineages detected in this study. To provide a broader evolutionary context to the haemosporidian lineages that we found, we also extracted all haemosporidian lineages that have been previously detected in Alaska based on the MalAvi “hosts and sites table” (Bensch et al. 2009), and included them in the analysis. We aligned all sequences using the Clustal Omega algorithm implemented in Geneious, and a maximum likelihood phylogeny was estimated using IQ-TREE (Trifinopoulos et al. 2016) implementing automatic model selection and 1000 Ultrafast bootstrap iterations.

We also estimated a phylogenetic tree for trypanosomatids detected in this study. We integrated the sequences that we recovered here with the 18S sequence dataset from Galen et al. (2020), who surveyed avian trypanosomes in Alaska and Pennsylvania, U.S.A. We identified unique genotypes from the Galen et al. (2020) dataset and combined those with the genotypes that we sequenced for this study. As we recovered a sequence from the trypanosomatid genus *Zelonia* in our survey, we also conducted a search of GenBank for all previously sequenced *Zelonia* isolates that overlapped the 18S fragment that we sequenced. All *Trypanosoma* and *Zelonia* sequences were aligned using the Clustal Omega algorithm implemented in Geneious, and a maximum likelihood phylogeny was estimated using IQ-TREE as described above.

## Results

### Haemosporidian prevalence and diversity

We tested for haemosporidian infection in 67 samples of 18 avian host species (Table 1). Of the 18 host species sampled, five had not previously been tested for haemosporidians using molecular methods according to the MalAvi database: *Sphyrapicus ruber*, *Empidonax difficilis*, *Poecile rufescens*, *Troglodytes pacificus*, and *Certhia americana*. Of the 67 samples, 28 (41.8%) were infected with at least one haemosporidian lineage, of which 17 samples were coinfections (25.4% coinfection prevalence; Table 1). Following sequencing, we found that 30 samples (45%) were infected with the genus *Haemoproteus*, 19 (28%) samples were infected with the genus *Leucocytozoon*, and there was only one sample that contained a parasite lineage of the genus *Plasmodium* (1.5% prevalence). Haemosporidian infections were concentrated in the families Turdidae (81.2% prevalence), Corvidae (77.8% prevalence), and Passerellidae (50% prevalence), while no individuals within the families that were sampled four or fewer times (Certhiidae, Phasianidae, Picidae, Regulidae, Trochilidae, Tyrannidae) were infected with any of the three genera (Table 1).

**Table 1:** Haemosporidian and trypanosomatid prevalence and diversity across 18 sampled host species from Prince of Wales Island, Alaska. For each host species that was sampled, the prevalence of every haemosporidian lineage that was found is listed separately. For trypanosomatids, overall prevalence is noted for each sampled host species

**Family**	**Species**	**Total hosts**	***Haemoproteus*** ** prevalence**	***Leucocytozoon*** ** prevalence**	***Plasmodium*** ** prevalence**	***Trypanosoma*** ** prevalence**
Certhiidae						
	*Certhia americana*	1	-	-	-	-
Corvidae						
	*Cyanocitta stelleri*	9	H_CATUST22 (1/9) H_CYASTE06 (3/9) H_CYASTE09 (4/9) H_CYACRI02 (1/9) H_TURDUS2 (1/9)	L_CATMIN06 (1/9) L_CYASTE10 (2/9)	-	2/9
Paridae						
	*Poecile rufescens*	9	H_TURDUS2 (1/9)	L_ZOONAE02 (1/9)	-	1/9
Parulidae						
	*Setophaga townsendi*	6	H_POEATR01 (1/6)	-	-	1/6 (*Zelonia sp.)*
	*Leiothlypis celata*	1	-	-	-	1/1 (*T. avium*)
Passerellidae						
	*Junco hyemalis*	4	H_JUHYE03 (1/4)	L_PERCAN01 (2/4)	-	1/4 (*T. avium*)
	*Melospiza lincolnii*	4	H_JUHYE03 (1/4)	L_ZOLEU02 (1/4) L_ZONLEU03 (1/4)	P_BT7 (1/4)	-
	*Melospiza melodia*	2	-	-	-	1/2
Phasianidae						
	*Falcipennis canadensis*	1	-	-	-	-
Picidae						
	*Sphyrapicus ruber*	4	-	-	-	-
Regulidae						
	*Regulus satrapa*	2	-	-	-	-
Trochilidae						
	*Selasphorus rufus*	1	-	-	-	-
Troglodytidae						
	*Troglodytes pacificus*	5	-	-	-	-
Turdidae						
	*Catharus guttatus*	10	H_POEATR01 (2/10)	L_ZOONAE02 (3/10) L_POEHUD01 (1/10) L_CATMIN01 (2/10) L_CATMIN05 (1/10) L_CATUST28 (1/10) L_PHYBOR02 (1/10)	-	-
	*Catharus ustulatus*	3	H_CATUST22 (2/3) H_TURDUS2 (1/3)	L_CATGUT02 (1/3) L_CATMIN06 (1/3)	-	-
	*Ixoreus naevius*	2	H_POEATR01 (1/2)	L_ZOONAE02 (1/2) L_CATMIN05 (1/2)	-	-
	*Turdus migratorius*	1	-	-	-	-
Tyrannidae						
	*Empidonax difficilis*	2	-	-	-	-

**Fig. 1:**
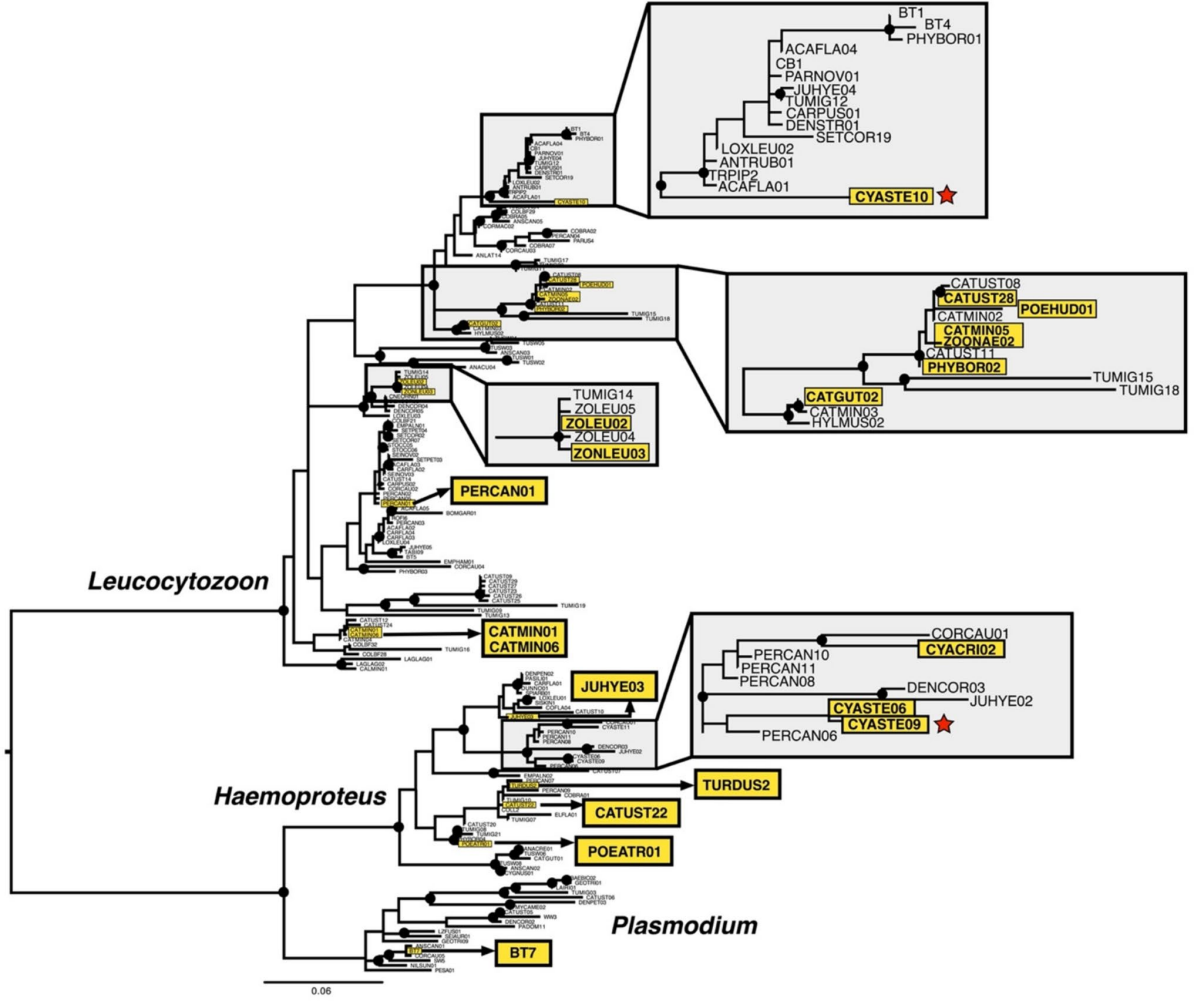
A maximum likelihood phylogeny of all haemosporidian *cytb* genetic lineages that have been detected in Alaska, U.S.A., including two novel lineages detected in this study. Clades in which haemosporidian lineages that were detected in this study are found are expanded; haplotypes found in this study are highlighted, and novel haplotypes are highlighted and denoted with a star. Nodes with ultrafast bootstrap support of ≥ 95 are labeled with a black circle

We detected 20 distinct haemosporidian genetic lineages, including seven *Haemoproteus*, 12 *Leucocytozoon*, and one *Plasmodium* lineage (Fig. 1). The most abundant lineage that we identified was L_ZOONAE02, which has been previously found only in host *Ixoreus naevius* in Alaska, though we recovered this lineage in three host species: *Catharus guttatus*, *Poecile rufescens*, and *Ixoreus naevius* (Table 1). The single *Plasmodium* lineage that we detected was BT7, which was isolated from *Melospiza lincolnii*. Two of the detected lineages were novel: the *Haemoproteus* lineage CYASTE09 isolated from *Cyanocitta stelleri*, and the *Leucocytozoon* lineage CYASTE10 also isolated from *Cyanocitta stelleri*. CYASTE09 was abundant in *Cyanocitta stelleri* (four of nine host samples) and was recovered in a clade of *Haemoproteus* that largely consisted of lineages that appear to nearly exclusively infect the avian family Corvidae (crows, jays, magpies, and ravens; Fig. 1). CYASTE10 was found to be sister to a clade of diverse *Leucocytozoon* lineages with no clear host affinities, including the generalist lineage CB1 which has been recorded from 45 host species (Fig. 1).

### Trypanosomatid prevalence and diversity

**Fig. 2:**
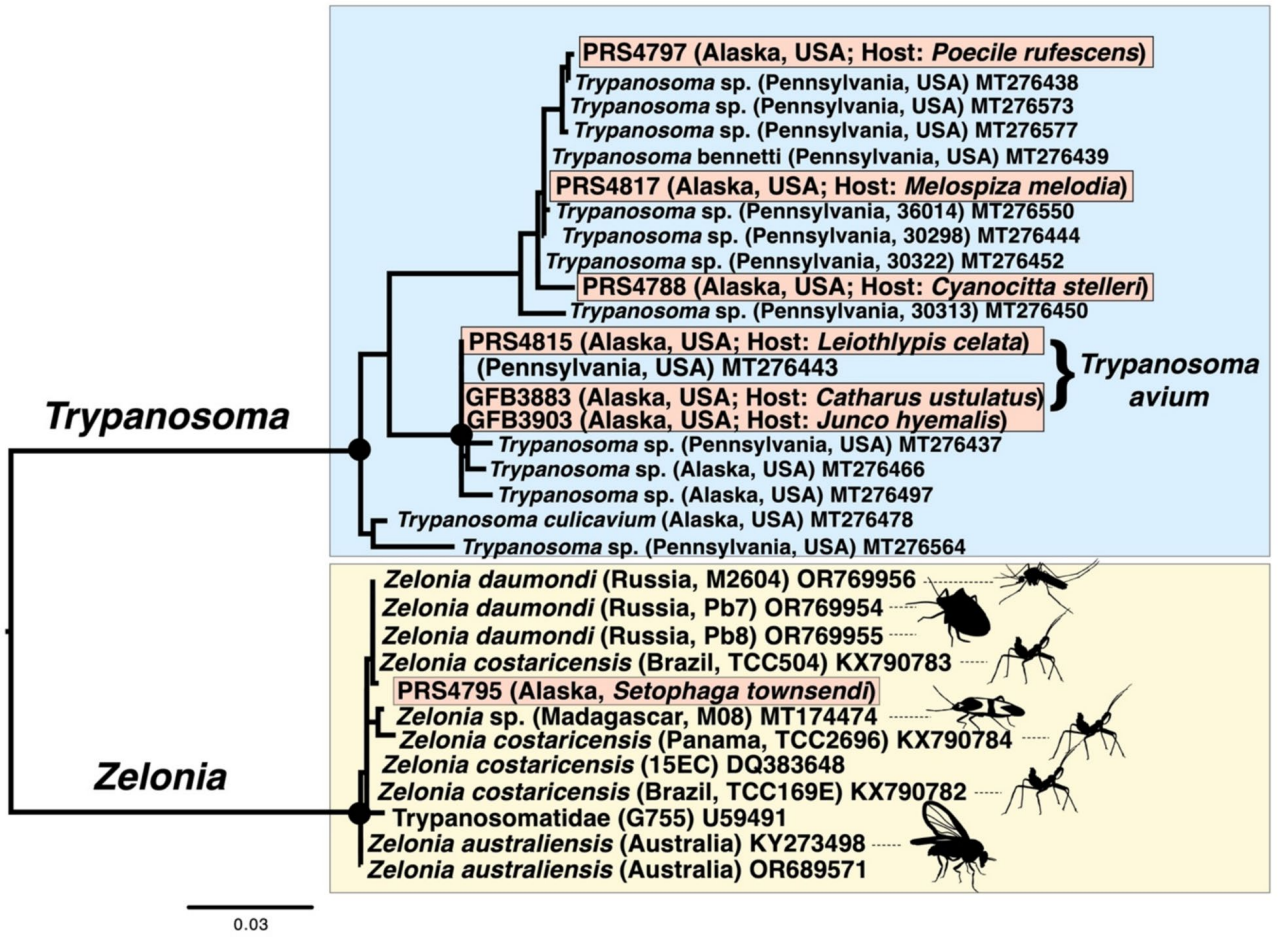
A maximum likelihood phylogeny of trypanosomatid 18S rRNA sequences. Genotypes detected in this study are labeled within red boxes; other included sequences were obtained from GenBank including all unique genotypes from Galen et al. (2020), and all previously sequenced *Zelonia* isolates. For *Zelonia* isolates, the host species from which the isolate was detected is represented graphically (if host species was recorded). All host images were obtained from www.phylopic.org. Nodes with ultrafast bootstrap support of ≥ 95 are labeled with a black circle

We detected a trypanosomatid in 8 of 67 host samples (12% prevalence). We obtained a sequence from seven of the eight positive amplifications, yielding five unique sequences. Three sequences had 100% BLAST identities to isolates previously identified as *Trypanosoma avium*. Three additional sequences were novel, and clustered in a clade consisting mostly of *Trypanosoma* genotypes that were isolated from Pennsylvania, U.S.A. (Fig. 2).

We found one trypanosomatid sequence that was obtained from host species *Setophaga townsendi* that was a close BLAST hit (one base pair difference) to the trypanosomatid *Zelonia daumondi*. A phylogenetic analysis that included all previously sequenced *Zelonia* isolates revealed little topological structure, though the novel isolate that we sequenced formed a clade with *Z. daumondi* and an isolate from Madagascar to the exclusion of *Z. costaricensis* and *Z. australiensis* (Fig. 2).

## Discussion

Studies of parasite biodiversity in high-latitude regions of the world are becoming increasingly important given recent changes in global temperatures (Miranda Paez et al. 2022). Temperature alterations can lead to changes in host distributions and migration patterns, which can alter the transmission and distribution of parasite species, potentially resulting in significant ecological consequences (Cizauskas et al. 2017). In our survey of blood parasites at a previously unstudied site, we found 20 genetic lineages of haemosporidians including two lineages that had not been discovered in previous studies. Haemosporidian prevalence varied widely among bird families, and the prevalence of *Plasmodium* infections was lower than previous studies in Alaska. Furthermore, we identified novel trypanosomatid genetic diversity, including an anomalous detection of a parasite in the genus *Zelonia* from bird tissue which represents the first record of this genus in the Nearctic region. These findings show that studying previously under-surveyed areas is likely to continue to reveal new parasite biodiversity and demonstrates the importance of surveying the parasite biodiversity of unstudied regions globally, particularly at high latitudes.

Our survey of avian blood parasites included five host species that have not previously been sampled for haemosporidians using genetics according to the MalAvi database (Bensch et al. 2009), as well as several other bird species for which very few samples have previously been studied. In general, molecular surveys of haemosporidians have revealed that expanding host species sampling leads to the discovery of novel haemosporidian genetic lineages, and our survey of a previously unsampled site in Alaska was no exception. Two new haemosporidian lineages were discovered, both found exclusively in the Steller’s jay, *Cyanocitta stelleri*. This host species has previously been found to host a high richness of haemosporidians in the genera *Haemoproteus* and *Leucocytozoon*, as eight lineages have been previously reported from just 28 *C. stelleri* samples as of January 2025 according to the MalAvi database. The high prevalence of new, host-specific lineages in *C. stelleri* indicates that further sampling of this host species across its broad distribution in western North America will likely reveal additional haemosporidian richness. Among the species that we sampled that had not been sampled previously, four species yielded no haemosporidian infections: *Certhia americana*, *Empidonax dificilis*, *Sphyrapicus ruber*, and *Troglodytes pacificus*. Though none of these host species were infected in our study, the sample sizes were low for each species. Increasing the sample sizes of host species that have been understudied for haemosporidian research should be emphasized in future studies, to more completely characterize haemosporidian biodiversity and infection patterns in North American and globally.

This study also adds to a wider picture that is building of haemosporidian abundance across space and host groups in Alaska. Though previous surveys have found that haemosporidians are highly prevalent in Alaskan birds (Oakgrove et al. 2014, 53% prevalence; Galen et al. 2019, 89% prevalence; De Amaral et al. 2023, 88% prevalence), we were surprised to find an overall prevalence of just over 40% on Prince of Wales Island. Though we noted a lower overall haemosporidian prevalence than in other previously studied regions of Alaska, we observed that several avian families were highly parasitized as has been found in other surveys. The family Turdidae had the highest overall prevalence of infections, followed closely by the family Corvidae (in our sample represented exclusively by *C. stelleri*). The high abundance of haemosporidians in the families Turdidae and Corvidae follows closely from several recent molecular surveys conducted in North America (Barrow et al. 2021, Galen et al. 2024). Particularly noteworthy was the near absence of *Plasmodium* in our survey, as *Plasmodium* has been found at higher prevalence in most haemosporidian studies in Alaska compared to the 1.5% prevalence in our analysis. For instance, Smith et al. (2016) found an overall *Plasmodium* infection prevalence of 9% in sampled grouse and ptarmigan in Alaska, while Oakgrove et al. (2014) recovered a *Plasmodium* prevalence of 7% from a community-wide survey of Alaskan birds. At present, it is difficult to determine whether our observed lack of *Plasmodium* can be attributed to chance, or whether there are biotic or abiotic factors driving this pattern. Of possible significance is the observation that Prince of Wales Island is a coastal temperate rainforest ecosystem, while previous haemosporidian surveys from Alaska have focused largely on interior boreal forest ecosystems. However, the impact of temperate rainforest habitats on mosquito abundance, diversity, and capacity to transmit haemosporidians does not appear to have been studied before and so we cannot speculate further. Similarly, while our analysis builds on a growing understanding of haemosporidian diversity and abundance at high latitudes in North America, the haemosporidian communities of other high-latitude regions throughout the world (e.g., Yusupova et al. 2023) are less well known and are in need of future attention.

We also surveyed for trypanosomatids using a molecular approach, which are rarely tested for in North American birds despite their abundance. The majority of trypanosomatid sequences that we generated were identical to a genotype that was previously identified as *T. avium* and has been found previously to be abundant not only in North America, but globally as well (Galen et al. 2020). The remaining *Trypanosoma* sequences that we generated have not been found before, and their species identities remain uncertain. Unfortunately, we were unable to characterize the morphology of any of the *Trypanosoma* isolates that we sequenced as we used avian tissue (muscle and liver) to detect these parasites. Using tissue samples for molecular surveys of blood parasites such as trypanosomes is convenient and can be effective (Williams et al. 2009), though it is not known whether tissue samples and blood samples are equally effective at characterizing avian trypanosome abundance and diversity. We confirm here that avian trypanosomes are capable of being detected and genetically characterized from host tissues using a conventional PCR approach, emphasizing the potential for this approach to be used with avian tissues archived in biodiversity repositories throughout the world.

Our discovery of a novel genotype of a trypanosomatid in the genus *Zelonia* was perplexing. *Zelonia* are monoxenous parasites of insects, while we amplified and sequenced material from this genus from an avian tissue sample. It is impossible to determine exactly how genetic material from *Zelonia* came to be present in avian tissue, though the most parsimonious explanation given our current knowledge of this group is that our detection was not due to a biological interaction but rather some form of environmental contamination from an infected insect (e.g., an insect infected with *Zelonia* could have contacted a tissue sample as it was being processed in the field). Alternatively, the observation that some trypanosomatids can be transmitted through oral ingestion (Votýpka and Svobodová 2004) is intriguing in light of our discovery, though there is currently no evidence that *Zelonia* can survive in a vertebrate host. Importantly, no *Zelonia* isolates have been amplified in the laboratory in which this work took place, so PCR contamination cannot account for this finding either. While we cannot determine the host source of the *Zelonia* genetic material that we detected, it does not change the fact that our study marks the first detection of *Zelonia* in the entire Nearctic region. Thus far, *Zelonia* has been detected largely in warm regions of the southern hemisphere (Barratt et al. 2017, Votýpka et al. 2020, Tullume-Vergara et al. 2023), though the discovery of *Z. daumondi* in Russia revealed that this genus is likely to be broadly distributed across the northern hemisphere as well into temperate and boreal regions of the world. Here, we confirmed that *Zelonia* is present at high latitudes in the Nearctic, though at present we cannot determine its insect host associations in this region.

In conclusion, our survey of avian parasites in an understudied island ecosystem in southern Alaska discovered novel haemosporidian and trypanosomatid diversity that enhances our understanding of the abundance and biogeography of these groups. Our survey suggests that in addition to undersurveyed areas, further studies focused on specific bird families will likely contribute to increased knowledge of blood parasite diversity and distributions. Lastly, our study emphasizes the need for dramatically expanded host and geographic sampling for understudied trypanosomatid groups such as *Zelonia*.

## Supplementary Information

Below is the link to the electronic supplementary material.

ESM 1 (XLSX 20.3 KB)

## Data Availability

DNA sequence data generated by this study have been deposited in the National Center for Biotechnology Information (NCBI) GenBank database (PV220920-PV220926 and PV232038-PV232039). Host-parasite interaction data was submitted to the MalAvi database, and can be found in supplementary information Appendix 1.
